# Enhanced Mechanical and Electromagnetic Shielding Properties of Mg Matrix Layered Composites Reinforced with Hybrid Graphene Nanosheet (GNS)–Carbon Nanotube (CNT) Networks

**DOI:** 10.3390/ma18153455

**Published:** 2025-07-23

**Authors:** Hailong Shi, Jiancheng Zhao, Zhenming Sun, Xiaojun Wang, Xiaoshi Hu, Xuejian Li, Chao Xu, Weimin Gan, Chao Ding

**Affiliations:** 1 Hunan Rongtuo New Material Research Co., Ltd., Xiangtan 411201, China; hailongshi@hit.edu.cn; 2 National Key Laboratory for Precision Hot Processing of Metals, Harbin Institute of Technology, Harbin 150001, China; 23s109188@stu.hit.edu.cn (J.Z.); sunzhenming611@163.com (Z.S.); huxiaoshi@hit.edu.cn (X.H.); 3 School of Material Science and Engineering, Harbin Institute of Technology, Harbin 150001, China; cxu@hit.edu.cn; 4 GEMS at Heinz Maier-Leibnitz Zentrum (MLZ), Helmholtz-Zentrum Hereon, Lichtenbergstr. 1, D-85748 Garching, Germany; weimin.gan@hereon.de; 5 Institute of High Energy Physics, Chinese Academy of Sciences, Beijing 100049, China; dingchao@ihep.ac.cn; 6 Spallation Neutron Source Science Center, Dongguan 523803, China

**Keywords:** Mg matrix composites, electromagnetic interference (EMI) shielding, graphene nanosheet (GNS), carbon nanotube (CNT), mechanical strengthening

## Abstract

The development of lightweight composites with superior mechanical properties and electromagnetic interference (EMI) shielding performance is essential for various structural and functional applications. This study investigates the effect of hybrid nanocarbon (graphene nanosheet (GNS) and carbon nanotube (CNT)) reinforcements on the properties of magnesium (Mg) matrix composites. Specifically, the GNS-CNT hybrid, which forms a three-dimensional interconnected network structure, was analyzed and compared to composites reinforced with only GNSs or CNTs. The objective was to determine the benefits of hybrid reinforcements on the mechanical strength and EMI shielding capability of the composites. The results indicated that the GNS-CNT/Mg composite, at a nanocarbon content of 0.5 wt.% and a GNS-CNT ratio of 1:2, achieved optimal performance, with a 55% increase in tensile strength and an EMI shielding effectiveness of 70 dB. The observed enhancements can be attributed to several key mechanisms: effective load transfer, which promotes tensile twinning, along with improved impedance matching and multiple internal reflections within the GNS-CNT network, which enhance absorption loss. These significant improvements position the composite as a promising candidate for advanced applications requiring high strength, toughness, and efficient electromagnetic shielding, providing valuable insights into the design of high-performance lightweight materials.

## 1. Introduction

With the rapid advancement of electronic technology, wireless communication devices have become indispensable not only in fields such as aerospace and military but also in everyday civilian life. In modern warfare, electronic warfare capabilities are increasingly pivotal, often determining the strategic advantage in conflicts. Furthermore, in daily life, electromagnetic radiation generated by electronic signal transmissions poses potential health risks, particularly to vulnerable groups such as children. As a result, electromagnetic interference (EMI) shielding has gained growing importance, serving not only to safeguard information security but also to protect public health [1,2].

Currently, mainstream electromagnetic shielding materials include metals such as copper, nickel, and silver [3,4], as well as non-metallic materials like graphite, graphene, MXene, and conductive resins [5,6,7,8,9]. Pure metallic materials primarily enhance electromagnetic interference (EMI) shielding through reflection of electromagnetic waves [10,11], whereas non-metallic materials improve shielding effectiveness by absorbing electromagnetic waves (EMWs) [12,13]. Each category of material has its own distinct advantages and limitations. Metallic materials, while highly effective in EMI reflection, are often characterized by high density and lack the ability to absorb EMWs, which can lead to secondary electromagnetic pollution and limit their applicability in stealth technology [14]. Conversely, non-metallic materials, despite their ability to absorb electromagnetic radiation, are generally constrained by insufficient mechanical strength, which limits their use as structural components [15].

As the lightest structural metal, magnesium (Mg) alloys offer numerous advantages, including high specific stiffness, high specific strength, low density, and ease of recycling, along with notable electromagnetic shielding properties [16,17]. However, their EMI shielding effectiveness is relatively high only within the low-frequency range and still primarily depends on electromagnetic wave reflection [7]. Recent studies have demonstrated that the fabrication of composite materials by introducing highly conductive reinforcements such as graphene nanosheets (GNSs) and carbon nanotubes (CNTs) into metal or polymer matrices can significantly enhance electromagnetic wave absorption, thereby improving the overall EMI shielding performance of these materials [18,19,20]. This approach is also applicable to Mg alloys. Additionally, pure Mg does not chemically react with materials like graphene or carbon nanotubes at high temperatures, providing a unique advantage for producing Mg-based composites through liquid-state processing methods such as stir casting [21].

In addition to optimizing material composition to enhance electromagnetic shielding performance, extensive research has also focused on improving shielding effectiveness through structural design, including mesh, layered, and porous structures [13,22,23,24]. Such structural designs not only improve impedance matching but also introduce numerous interfaces within the composite, causing electromagnetic waves penetrating the material to undergo multiple internal scattering, thereby achieving enhanced shielding effectiveness. As a two-dimensional material (a material in which electrons can only move freely (plane motion) on non-nanometer scales (1–100 nm) in two dimensions), graphene nanosheets (GNSs) are particularly well-suited for incorporation into Mg matrices to produce GNS/Mg layered composites, owing to their inherent structural properties. However, GNSs have a natural tendency to restack (due to the ultra-high specific surface area and aspect ratio, strong Van der Waals forces exist between GNSs), which can lead to interlayer cracking in the composite, ultimately resulting in premature failure. Introducing nanoscale particles between the graphene layers could facilitate the integration of multiple graphene layers while preserving the mechanical integrity of the composite (the nanoparticle–graphene matrix can form an interconnected three-dimensional network structure, and the adjacent GNSs are bridged by CNTs like extended tentacles, thus promoting the integration of multiple graphene layers). Additionally, constructing a graphene mesh structure within the composite material could further enhance its electromagnetic shielding performance [25].

Due to the ultra-high specific surface area and aspect ratio of graphene and carbon nanotubes, there are strong Van der Waals forces between them. As a result, nanocarbon materials tend to agglomerate when only graphene or carbon nanotubes are used as reinforcement to synthesize layered Mg matrix composites. The stacked graphene or carbon nanotubes form a thick graphene film or carbon nanotube film on the surface of the Mg foil, which hinders the thermal diffusion bonding between adjacent layer elements during subsequent hot-press sintering [6,7]. However, by hybridizing graphene and carbon nanotubes, GNSs and CNTs can form a bridge between each other, ultimately creating an interconnected three-dimensional network. The adjacent GNSs are bridged by CNTs like extended tentacles, while the GNSs within the interlayer effectively prevent the stacking and agglomeration of CNTs.

In light of the aforementioned considerations, we propose the use of spray deposition followed by vacuum hot-press sintering to introduce nanocarbon materials, namely graphene nanosheets (GNSs) and carbon nanotubes (CNTs), into a Mg matrix to fabricate layered GNS-CNT/Mg composites. This approach aims to synergistically enhance both the mechanical properties and the electromagnetic shielding effectiveness of the Mg matrix. Specifically, by alternately spray-depositing CNTs and GNSs onto Mg foils, we create a reinforcing nanolayer with a network structure, wherein the Mg foil acts as the micron-scale layer. A systematic characterization of the microstructure, mechanical properties, and electromagnetic performance of the fabricated composites was conducted. Additionally, a detailed analysis of the underlying mechanisms responsible for the enhancements in both the mechanical and the electromagnetic shielding properties due to the unique structural features of these composites was performed. This study provides valuable insights into the design of high-performance electromagnetic shielding materials, particularly those requiring enhanced electromagnetic wave absorption and energy dissipation.

## 2. Experimental Procedure

### 2.1. Raw Materials

Cold-rolled pure Mg foils with a thickness of 50 μm were used as the matrix material in this study (purchased from Beijing Qiansuo Nonferrous Metal Products Co., Ltd., Beijing, China). The chemical composition of the initial Mg foils is provided in Table 1. The reinforcement materials comprised two components: multiwalled carbon nanotubes (MWCNTs) with a diameter of 30–80 nm and a length of ≤10 μm (supplied by Chengdu Institute of Organic Chemistry, Chinese Academy of Sciences, Chengdu, China), and graphene nanosheets (GNSs) with a diameter of 0.5–5 μm and a thickness of 0.8–1.2 nm (purchased from Nanjing Xianfeng Nanomaterial Technology Co., Ltd., Nanjing, China).

### 2.2. Fabrication Procedure of the GNS-CNT/Mg Layered Composites

**Figure 1:**
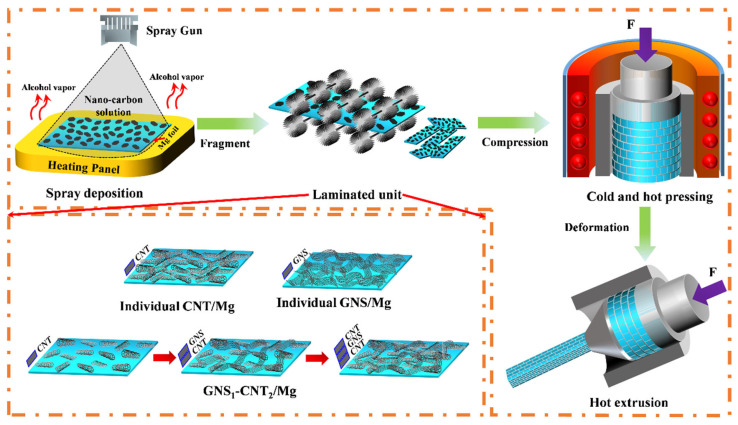
Schematic diagram of the fabrication process of GNS-CNT/Mg layered composites.

Figure 1 illustrates the preparation process of the layered composites, which consisted of three main steps: spray deposition (SPD), vacuum hot-press sintering, and hot extrusion. In the spray deposition process, carbon nanotubes (CNTs) and graphene nanosheets (GNSs) were uniformly deposited onto the surface of the Mg foil to form a nanocarbon/Mg layered basic unit. Before spray deposition, CNTs and GNSs were separately dispersed in ethanol to prepare suspensions with concentrations of 0.3 g/L and 0.6 g/L, respectively. The suspensions were then treated by ultrasonic agitation for 12 h. Additionally, the initial Mg foil was polished using 350# sandpaper to remove the oxide layer on the surface. A “sandwich” structure was formed on the surface of the Mg foil by sequentially spray depositing CNT-GNS-CNT, with GNSs as the middle layer and CNTs on both sides as reinforcing layers. The spray deposition was carried out using a high-atomization spray gun at a deposition pressure of 0.3 MPa and a spray height of 30 cm, employing a scanning spray deposition method. The content of each reinforcing material in the composite was controlled by adjusting the deposition volume of the CNT and GNS solutions.

The deposited Mg foil was then crushed into 2 mm × 2 mm rectangular pieces and sintered in a vacuum hot-press furnace at 630 °C under a pressure of 50 MPa for 6 h. To densify the composite, the sintered material was hot-extruded at an extrusion temperature of 400 °C, an extrusion speed of 0.1 mm/s, and an extrusion ratio of 29:1. For comparison, pure bulk Mg and layered pure Mg were also prepared using the same method. Subsequently, using a method similar to flake powder metallurgy, the composite sheets were fragmented and then utilized to fabricate GNS-CNT/Mg layered composites through vacuum hot-press sintering followed by hot extrusion. GNS-CNT/Mg layered composites went through vacuum hot-press sintering followed by hot extrusion.

### 2.3. Characterization

A field emission scanning electron microscope (SEM, SUPRA 55 SAPPHIRE, ZEISS, Oberkohen, Germany) was used to observe the dispersion of GNS-CNT on the Mg foil surface and the fracture morphology of the GNS-CNT/Mg composite. The microstructure of the GNS-CNT/Mg composite in the extrusion direction (ED) was analyzed using electron backscatter diffraction (EBSD, SUPRA 55 SAPPHIRE, ZEISS, Oberkohen, Germany). The interface of the GNS-CNT/Mg was characterized by transmission electron microscopy (TEM, Talos F200x, ThermoFisher Scientific, Portland, OR, USA).

The tensile test was conducted using an electronic universal testing machine (Instron 5569, Instron, Boston, MA, USA) in accordance with the ASTM: E8/E8m-13a standard [26], with a crosshead speed of 0.5 mm/min. The dimensions of the tensile specimen were a gauge length of 15 mm and a width of 6 mm.

The electromagnetic interference shielding effectiveness (EMI SE) of the GNS-CNT/Mg composite was measured in the X-band (8.2–12.4 GHz) using the waveguide method with a vector network analyzer (VNA) (N5227A, Agilent, Palo Alto, CA, USA). Rectangular CNT/Mg composite specimens were processed to dimensions of 22.86 mm × 10.16 mm × 5 mm for EMI SE measurements. A digital conductivity meter (Sigma 2008B, Day Research Instruments, Tianyan Instrument, Xiamen, China) was used to measure the conductivity of specimens with dimensions of φ10 mm × 1 mm.

## 3. Results

### 3.1. Alternating Spray Deposition of GNS-CNT Layer

**Figure 2:**
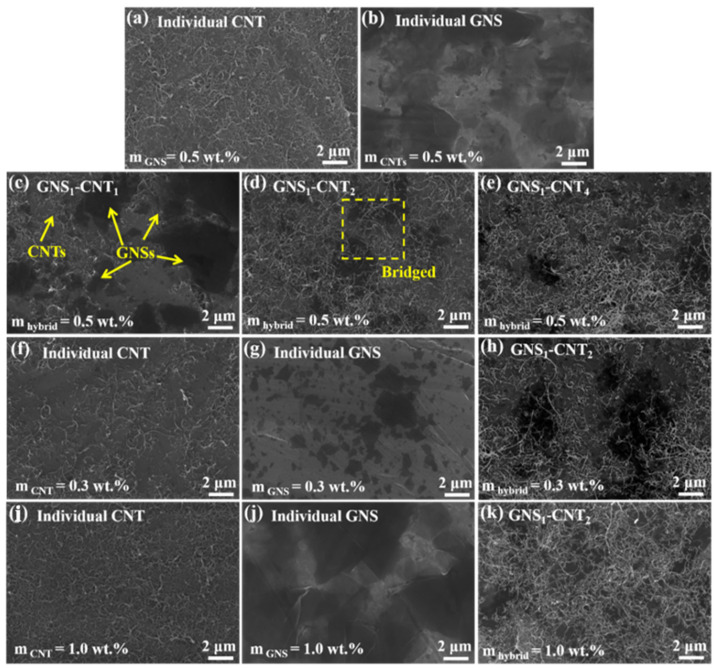
SEM images showing the distribution of nanocarbon on the Mg foil surface at various mass fractions: (**a**–**e**) distribution morphology of CNTs, GNSs, GNS_1_-CNT_2_, GNS_1_-CNT_2_, and GNS_1_-CNT_4_ at a total nanocarbon content of 0.5 wt.%; (**f**–**h**) distribution morphology of CNTs, GNSs, and GNS_1_-CNT_2_ at a total nanocarbon content of 0.3 wt.%; (**i**–**k**) distribution morphology of CNTs, GNSs, and GNS_1_-CNT_2_ at a total nanocarbon content of 1.0 wt.%.

Based on prior experimental experience, this study initially constructed GNS-CNT reinforcements with different GNS-CNT ratios, maintaining a total nanocarbon content of 0.5 wt.%, as shown in Figure 2a–e. It was observed that the distribution morphology of GNS-CNT significantly differed from that of GNSs or CNTs alone with the same nanocarbon content. In the GNS-CNT layer, separately distributed GNSs and CNTs acted as bridges, ultimately forming an interconnected three-dimensional network structure.

**Figure 3:**
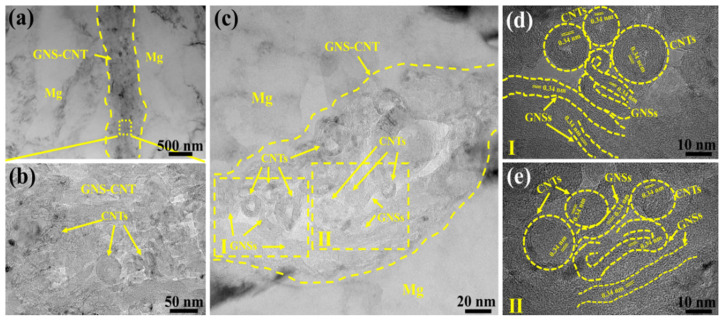
TEM images of as-extruded GNS-CNT/Mg layered composites: (**a**) interface between Mg matrix and the reinforcements, (**b**) higher magnification of the area circled with dotted yellow lines in (**a**), (**c**) hybrid GNS-CNT reinforcements within the composite, (**d**,**e**) high-resolution TEM images corresponding to areas circled in (**c**).

Notably, when the mass ratio between GNSs and CNTs was 1:1 (denoted as GNS_1_-CNT_1_), the CNT content was relatively low, resulting in insufficient CNTs to bridge adjacent GNSs, leaving large exposed areas of the Mg foil surface between adjacent GNSs, as depicted in Figure 2c. Conversely, when the mass ratio between GNSs and CNTs was 1:4, the low GNS content led to insufficient GNSs to effectively hinder the stacking and agglomeration of excess CNTs, as shown in Figure 2e. When the GNS-CNT mass ratio was 1:2, a well-developed three-dimensional network structure was formed on the Mg foil surface, where CNTs bridged adjacent GNSs like extended antennae, and the GNSs located in the interlayer effectively inhibited CNT stacking and agglomeration, as illustrated in Figure 3d.

Subsequent experiments revealed that at a total nanocarbon content of 0.5 wt.%, GNS_1_-CNT_2_ exhibited a more significant synergistic strengthening effect on the mechanical properties and electromagnetic shielding performance of the Mg matrix compared to the other two GNS-CNT ratios. To verify the generality of this synergistic effect, this study also investigated the influence of GNS_1_-CNT_2_ on the mechanical properties and electromagnetic shielding efficiency of the Mg matrix at total nanocarbon contents of 0.3 wt.% and 1.0 wt.% compared to CNTs and GNSs at the same content. Figure 2f–k show the corresponding distribution morphology of nanocarbon on the surface of the layered unit. When the total nanocarbon content was 0.3 wt.%, the reinforcement content was low, resulting in many nanocarbon-depleted regions on the Mg foil surface, which could not effectively utilize the reinforcement potential of the GNS_1_-CNT_2_ three-dimensional network, as shown in Figure 2f–h. When the total nanocarbon content was 1.0 wt.%, the excessive CNTs and GNSs nearly covered the entire Mg foil surface, hindering the subsequent sintering bonding of adjacent Mg foils. However, GNS_1_-CNT_2_, with its three-dimensional network structure, alleviated this phenomenon to some extent, inhibiting the stacking and agglomeration tendencies of GNSs and CNTs when used as reinforcements, as shown in Figure 2j–k.

Figure 3 shows the TEM images of the GNS-CNT/Mg composite at the interlayer interface of the GNS_1_-CNT_2_/Mg layered composite at a nanocarbon content of 0.5 wt.%. As seen in Figure 3a, GNS-CNT exhibited a continuous layered distribution, being tightly bonded to the Mg matrix without any noticeable defects. Upon further magnification of the hybrid structure within the layer, the clearly hollow ring-like structures in the GNS-CNT layer can be explicitly identified as carbon nanotubes (CNTs). However, other regions with various curved sheet-like structures could correspond either to the two-dimensional graphene sheets or to the tube walls of CNTs, making it difficult to accurately determine the exact reinforcing material, as shown in Figure 3b.

To accurately characterize the hybrid structure within the GNS-CNT layer, two regions with pronounced hybrid structures were selected for high-resolution transmission electron microscopy (HRTEM) observation at higher magnification and clarity, as shown in Figure 3c–e. In the HRTEM image corresponding to region I in Figure 3c, the GNSs located between CNTs effectively inhibited CNT stacking and agglomeration, thereby promoting good interfacial bonding, as shown in Figure 3d. In region II, adjacent GNSs were bridged by CNTs like extended antennae, while adjacent CNTs were also connected through the overlapping graphene, thereby forming a unique three-dimensional network structure, as shown in Figure 3e. Moreover, after undergoing intense hot extrusion, and despite prolonged ion thinning of the transmission specimen, the interlayer GNSs and CNTs in the GNS_1_-CNT_2_/Mg layered composite still maintained the integrity of the three-dimensional network structure, further indicating the strong bonding between GNSs and CNTs within the GNS-CNT reinforcement.

**Figure 4:**
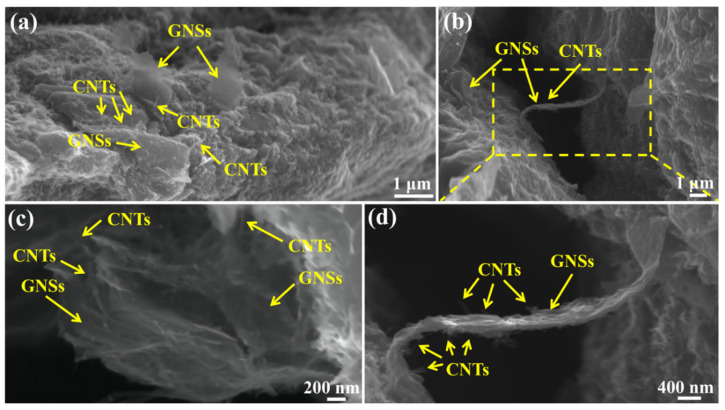
SEM images of the fracture surfaces in the GNS-CNT/Mg layered composites: (**a**,**b**) pulled-out GNSs and CNTs at the fracture surface, (**c**,**d**) CNT-GNS network at the fracture surface.

In addition to using TEM to observe the hybrid structure of GNS-CNT reinforcements at the interface of the layered composite, the hybrid characteristics of GNS-CNT were also directly observed at the tensile fracture. Figure 4 shows the fracture morphology of the tensile specimen of the 0.5 wt.% GNS_1_-CNT_2_/Mg layered composite. After tensile deformation, the fracture still displayed the intact three-dimensional network structure, further indicating the strong bonding between CNTs and GNSs. Figure 4a,c show the pull-out phenomenon of the GNS-CNT reinforcement at the fracture surface. Compared to using GNSs or CNTs alone as reinforcements, the strong bonding between GNSs and CNTs in the GNS-CNT reinforcement meant that, during fracture failure and deformation of the composite, energy was expended not only to overcome the friction between the reinforcement and the Mg matrix but also to break the bonding forces between GNSs and CNTs. This resulted in higher energy consumption, which effectively reduced the crack propagation rate. Figure 4b,d present the bridging phenomenon of the GNS-CNT reinforcement at the fracture, where CNTs acted like “rivets,” pinning one end of the GNSs to the Mg side. This contributed to enhanced interfacial bonding strength between the reinforcement and the matrix, increasing the energy required for fracture failure of the Mg matrix, thereby enhancing its mechanical properties.

### 3.2. Mechanical Properties of Pure Mg and the CNT-GNS Composites

**Figure 5:**
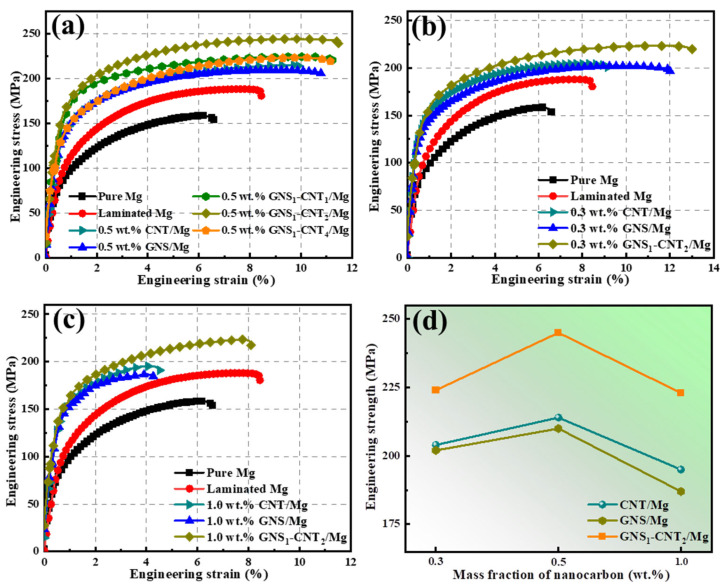
Engineering stress–strain curves of pure Mg and composites reinforced by CNTs alone, GNSs alone, and GNS-CNT: (**a**) GNS-CNT/Mg composites with reinforcement mass fraction of 0.5% and different GNS-CNT ratios, (**b**) mass fraction of nanocarbon reinforcements of 0.5%, (**c**) mass fraction of nanocarbon reinforcements of 0.5%, (**d**) comparison of strength for CNT/Mg, GNS/Mg, and GNS-CNT/Mg with different reinforcement contents.

Figure 5 shows the mechanical performance curves of layered Mg-based composites reinforced with CNT, GNS, and GNS-CNT nanocarbon reinforcements. Specific values are listed in Table 2. As illustrated in Figure 5a, the tensile strength of CNT/Mg (214 MPa) and GNS/Mg (210 MPa) composites increased by 35% and 33%, respectively, compared to pure Mg. Compared to using GNSs or CNTs alone as reinforcements, the three-dimensional network structure formed by hybridizing GNSs and CNTs in different ratios demonstrated a significant strengthening and toughening effect, resulting in improved mechanical properties of the composites. Among them, the GNS_1_-CNT_2_ reinforcement exhibited the most pronounced strengthening and toughening advantage, providing the GNS_1_-CNT_2_/Mg composite with a tensile strength of 245 MPa, which was 55% higher than that of pure Mg, while maintaining good elongation (11.5%).

The tensile strengths of GNS_1_-CNT_1_/Mg (224 MPa) and GNS_1_-CNT_4_/Mg (222 MPa) composites followed closely, showing increases of 42% and 40%, respectively, compared to pure Mg. In the case of GNS_1_-CNT_1_/Mg, the lower CNT content resulted in insufficient CNTs to bridge adjacent GNS sheets, leading to larger gaps in the three-dimensional network, with large exposed areas of the Mg foil between GNSs, which limited the effectiveness of the GNS-CNT network in strengthening, as shown in Figure 2c. In GNS_1_-CNT_4_/Mg, the high CNT content combined with a low GNS content meant that the limited amount of GNSs could not effectively prevent CNT stacking, resulting in poor interfacial bonding, as shown in Figure 2e.

The synergistic strengthening and toughening effect of GNS_1_-CNT_2_ was also verified in composites reinforced with other nanocarbon contents, as shown in Figure 5b–d. When the total nanocarbon content was 0.3 wt.%, the tensile strength of the GNS_1_-CNT_2_/Mg composite reached 224 MPa, which represents a 42% increase compared to pure Mg, while maintaining a high elongation of 13.1%. In contrast, CNT/Mg (204 MPa) and GNS/Mg (202 MPa) composites only showed improvements of 29% and 28%, respectively, as shown in Figure 5b. At a total nanocarbon content of 1.0 wt.%, the same trend was observed: the GNS_1_-CNT_2_/Mg composite exhibited a tensile strength of 223 MPa with an elongation of 8.1%, representing a 41% increase over pure Mg, whereas CNT/Mg (195 MPa) and GNS/Mg (187 MPa) composites only improved by 23% and 18%, respectively, as shown in Figure 5c. Additionally, the three-dimensional GNS_1_-CNT_2_ reinforcement showed a similar enhancement trend for elongation as for tensile strength, meaning that for the same nanocarbon content, the GNS_1_-CNT_2_/Mg composite demonstrated superior elongation.

By comparing the mechanical properties of CNT/Mg, GNS/Mg, and GNS_1_-CNT_2_/Mg systems at different nanocarbon contents, as shown in Figure 5d, it can be clearly seen that, at the same total nanocarbon content, the three-dimensional network GNS_1_-CNT_2_ reinforcement offered a distinct mechanical enhancement advantage over reinforcement with CNTs or GNSs alone. Additionally, the tensile strength of the 0.5 wt.% GNS_1_-CNT_2_/Mg layered composite was found to be optimal. When the reinforcement content was low (0.3 wt.%), a relatively complete three-dimensional network structure could not be effectively formed, and the limited amount of nanocarbon could not adequately enhance the mechanical properties of the composite. When the reinforcement content was too high (1.0 wt.%), the entire Mg foil surface was almost fully covered, which hindered subsequent thermal diffusion bonding between adjacent Mg layers. Therefore, further studies will focus on the strengthening and toughening mechanisms of the GNS_1_-CNT_2_/Mg layered composite with a total nanocarbon content of 0.5 wt.%.

### 3.3. Electromagnetic Interference Shielding Performance of Pure Mg and GNS-CNT/Mg Composites

The scattering parameters S_11_ and S_21_ were directly measured using a vector network analyzer. The reflection loss (S_ER_), absorption loss (S_EA_), and total shielding effectiveness (S_ET_) of the sample can be calculated using the following formulas [27]:

(1) $$R\;=\;{\left|S_{11}\right|}^{2}\;=\;{\left|S_{22}\right|}^{2},$$

(2) $$T={\left|S_{12}\right|}^{2}={\left|S_{21}\right|}^{2},$$

(3) A=1−R−T,

(4) $${\mathrm{SE}}_{R}=10\mathrm{lg}\frac{1}{1-R},$$

(5) $${\mathrm{SE}}_{A}=10\mathrm{lg}\frac{1-R}{T},$$

(6) $${\mathrm{SE}}_{T}={\;\mathrm{SE}}_{R}+{\mathrm{SE}}_{A},$$

where R, T, and A are the reflection, transmission, and absorption coefficients of the samples to electromagnetic waves.

**Figure 6:**
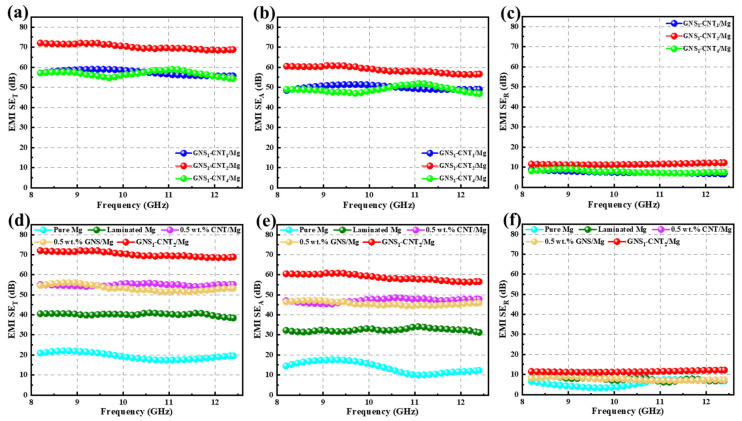
EMI SE of Mg and layered Mg matrix composites reinforced by various nanocarbon materials (nanocarbon content kept at 0.5 wt.%): (**a**–**c**) EMI SE of GNS-CNT/Mg with different GNS-CNT ratios, (**d**–**f**) EMI SE of pure Mg and Mg matrix composites reinforced with CNTs, GNSs, and GNS-CNT hybrid reinforcements with a mass fraction ratio of GNSs and CNTs as 1:2.

This study primarily compares the strengthening advantage of GNS-CNT reinforcements over GNSs and CNTs in terms of the electromagnetic shielding effectiveness (EMI SE) of the Mg matrix. Initially, the effect of three hybrid reinforcements—GNS_1_-CNT_1_, GNS_1_-CNT_2_, and GNS_1_-CNT_4_—on the EMI SE of the composite was investigated at a nanocarbon content of 0.5 wt.%, as shown in Figure 6a–c. As can be seen from the figure, GNS_1_-CNT_2_ showed the most significant improvement in the EMI SE of the Mg matrix compared to the other two hybrid reinforcements, with a total shielding effectiveness (S_ET_) of up to 70 dB. Subsequently, the EMI SE of the GNS_1_-CNT_2_/Mg layered composite was compared with the CNT/Mg and GNS/Mg layered composites at the same nanocarbon content, as shown in Figure 6d–f. The comparison reveals that the three-dimensional GNS_1_-CNT_2_ reinforcement had a significant advantage in improving the EMI SE of the Mg matrix compared to GNSs and CNTs. The total shielding effectiveness (S_ET_) of GNS_1_-CNT_2_/Mg was 25% and 21% higher than that of the GNS/Mg and CNT/Mg composites, respectively. Furthermore, Figure 6e,f shows that this advantage primarily came from the improved absorption loss (S_EA_) of the incident electromagnetic waves by the GNS_1_-CNT_2_ reinforcement, with almost no effect on reflection loss (S_ER_).

**Figure 7:**
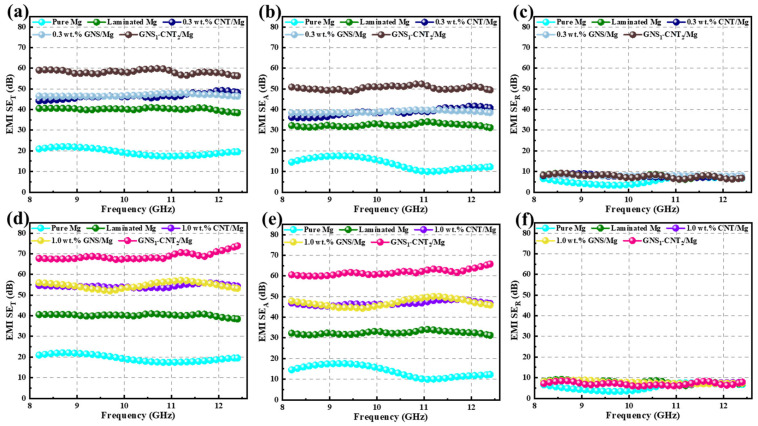
EMI shielding effectiveness (SE) of pure Mg and layered Mg matrix composites reinforced with various nanocarbon structures: (**a**–**c**) EMI SE of Mg matrix composites reinforced with GNSs, CNTs, and GNS-CNT at a reinforcement mass fraction of 0.3%, (**d**–**f**) EMI SE of Mg matrix composites reinforced with GNSs, CNTs, and GNS-CNT at a reinforcement mass fraction of 1.0%.

To further verify the advantage of the three-dimensional GNS_1_-CNT_2_ reinforcement in enhancing electromagnetic shielding effectiveness compared to CNTs and GNSs, GNS_1_-CNT_2_ was applied in layered shielding materials prepared with other nanocarbon contents, as shown in Figure 7. The results showed that the three-dimensional GNS_1_-CNT_2_ reinforcement also demonstrated a significant advantage in improving the electromagnetic shielding effectiveness of the Mg matrix at nanocarbon contents of 0.3 wt.% and 1.0 wt.% compared to using CNTs or GNSs alone as reinforcements. However, when the nanocarbon content was 0.3 wt.%, the shielding effectiveness of the GNS_1_-CNT_2_/Mg composite decreased to 60 dB due to the lower nanocarbon content. At a nanocarbon content of 1.0 wt.%, the shielding effectiveness of the GNS_1_-CNT_2_/Mg composite was similar to that at 0.5 wt.%, both reaching 70 dB. This indicates that the nanocarbon content of 0.5 wt.% was the threshold for maximizing the electromagnetic shielding performance of GNS_1_-CNT_2_ reinforcement in the Mg matrix. Some previous studies on improving electromagnetic shielding efficiency (in the X-band: 8.2–12.4 GHz) are summarized in Table 3. As shown in Table 3, compared to our previous work, the performance of the GNS-CNT hybrid as a reinforcement for electromagnetic shielding was significantly better than that of GNSs or CNTs alone. The composite achieved a 15 dB higher SE than composites reinforced with only CNTs or GNSs. Furthermore, when compared with other materials in this frequency range, the hybrid material still leads, providing greater SE improvement with a lower reinforcement mass fraction.

**Figure 8:**
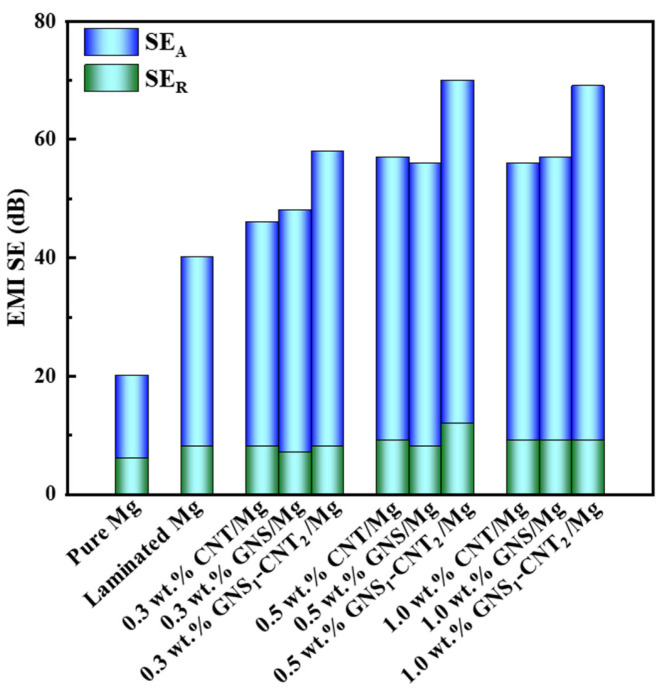
Comparison of EMI SE between Mg and nanocarbon/Mg layered composites.

A comparison of the electromagnetic shielding performance of pure Mg and various nanocarbon/Mg layered composites is shown in Figure 8. The improvement in shielding performance primarily resulted from enhanced absorption of incident electromagnetic waves, which was facilitated by the introduction of nanocarbon materials. At a nanocarbon content of 0.5 wt.%, the shielding performance reached a threshold. Notably, the three-dimensional GNS_1_-CNT_2_ reinforcement provided superior shielding effectiveness compared to CNTs or GNSs alone at the same nanocarbon content. From the performance comparison in Table 4, it is evident that the yield strength and tensile strength of the network-structured material with the GNS-CNT hybrid were approximately 30 MPa higher than those of the layered structure material with GNSs and CNTs alone. The elongation was similar, while the electromagnetic shielding performance was about 15 dB higher. Compared to other non-metallic composites, the mechanical properties of GNS_1_-CNT_2_/Mg were significantly better than those of the non-metallic material, while its electromagnetic shielding performance was about 20 dB higher. When compared with GO/AZ31 and GO-SnO_2_/AZ31, the electromagnetic shielding performance of GNS_1_-CNT_2_/Mg was much higher, with little difference in mechanical properties.

## 4. Discussion

### 4.1. Strengthening Mechanism of Layered GNS-CNT/Mg Composites

It is well established that Orowan strengthening, thermal mismatch strengthening, grain refinement, and load transfer are the primary strengthening mechanisms in traditional metal matrix composites. However, in this study, the distribution of nanocarbon reinforcements was predominantly at grain boundaries, rendering the Orowan strengthening effect negligible. Furthermore, previous research [6,7] has shown that introducing nanocarbon reinforcements between layers primarily promotes dynamic recrystallization at the interlayer interface, leading to recrystallized grains that account for only a small portion of the total grain volume. As a result, the impact of grain refinement on the overall average grain size is minimal, and grain refinement strengthening is largely insignificant.

In summary, thermal mismatch strengthening and load transfer strengthening are identified as the two main mechanisms contributing to the improved properties of the GNS_1_-CNT_2_/Mg layered composite. The GNS_1_-CNT_2_ reinforcement in this study primarily consisted of introducing GNS layers into the CNT interlayers, resulting in an overall “sandwich” structure, while maintaining a three-dimensional network at the microscopic level. The GNS content, being only half that of CNT, plays a critical role in preventing CNT stacking and promoting effective load transfer. This study mainly focuses on comparing the strengthening mechanisms between composites reinforced with CNTs alone and those with the three-dimensional GNS_1_-CNT_2_, highlighting the significant improvements in mechanical and electromagnetic properties achieved by using this hybrid reinforcement.

#### 4.1.1. Load Transfer Strengthening

Before performing the calculation, it is necessary to determine the relationship between the length of the reinforcement and its critical length. Specifically, for the GNS_1_-CNT_2_/Mg layered composite, the three-dimensional GNS-CNT network can only effectively contribute to load transfer when the stress is transmitted through the interface to the critical length of the reinforcement. Therefore, based on the Kelly–Tyson equation, the theoretical contribution of carbon nanotube load transfer to the yield strength (YS) of the reinforced composite (denoted as $∆\sigma_{\mathrm{LT}}$) can be expressed by the following formula [35]:

(7) $$∆\sigma_{\mathrm{LT}}\;={\;\sigma }_{\mathrm{re}}V_{\mathrm{re}}\left(\frac{l_{\mathrm{re}}}{2l_{c}}\right){-\;\sigma }_{\mathrm{ym}}V_{\mathrm{re}},\;\;\;\;\;\;l\;\leq {\;l}_{c}$$

(8) $$∆\sigma_{\mathrm{LT}}\;={\;\sigma }_{\mathrm{re}}V_{\mathrm{re}}\left(1-\frac{l_{\mathrm{re}}}{2l_{c}}\right)-{\;\sigma }_{\mathrm{ym}}V_{\mathrm{re}},\;\;\;\;\;\;l\;>{\;l}_{c}$$

where $\sigma_{re}$ is the strength of CNTs, $V_{re}$ is the volume fraction of CNTs, $l_{re}$ is the length of CNTs, and $l_{c}$ is the critical length of CNTs.

**Figure 9:**
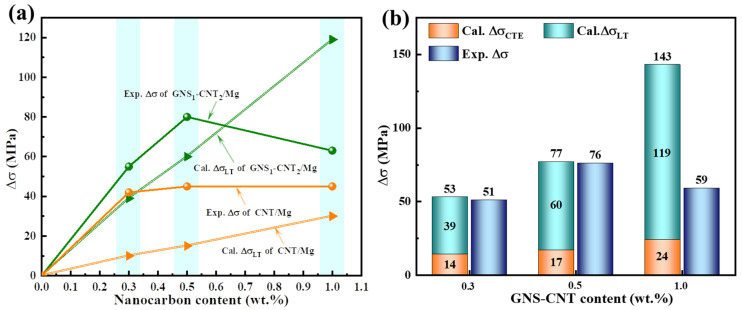
Theoretical and experimental yield strength increment of CNT/Mg and GNS_1_-CNT_2_/Mg layered composites: (**a**) experimental yield strength and predicted strength increment contributed by load transfer for CNT/Mg and GNS_1_-CNT_2_/Mg layered composites with varying nanocarbon content, (**b**) comparison of experimental yield strength of GNS_1_-CNT_2_/Mg layered composites contributed by different strengthening mechanisms.

Figure 9a shows the experimentally measured yield strength increment (Δ*σ*) and the predicted load transfer strengthening value ($\Delta \sigma_{\mathrm{LT}}$) for CNT/Mg and GNS_1_-CNT_2_/Mg layered composites as a function of increasing nanocarbon content. As seen in the figure, the overall trend indicates that the contribution of load transfer in both material systems increased linearly with the increase in nanocarbon content. By further comparing the slopes of the load transfer contribution curves for the two reinforcements, it is evident that the load transfer strengthening efficiency of GNS_1_-CNT_2_ in the Mg matrix was significantly higher than that of CNT. This suggests that the three-dimensional GNS-CNT reinforcement was more effective than the pure CNTs in facilitating load transfer at the interlayer interface, making load transfer strengthening the main mechanism contributing to the yield strength of the GNS-CNT/Mg composite.

The significant load transfer strengthening advantage of the GNS_1_-CNT_2_ reinforcement was primarily due to the complex three-dimensional network formed by the interconnection of GNSs and CNTs, which increased the effective size of the GNS-CNT reinforcement compared to that of pure CNTs. Reinforcements with a larger effective size have greater potential to enhance load transfer at the interface. Additionally, if the reinforcement is considered to be a spherical nanoparticle, the effective particle diameters of GNSs, CNTs, and GNS_1_-CNT_2_ reinforcements (denoted as $d_{p(GNS)}$, $d_{p(CNT)}$, and $d_{p({GNS}_{1}-{CNT}_{2})}$, respectively) can be calculated using Equations (9)–(11) [36,37,38]:

(9) $$d_{G}\;=\;{\left(\frac{3L^{2}t}{4\pi }\right)}^{1/3},$$

(10) $$d_{c}=\sqrt[{3}]{\frac{3Fl_{\mathrm{re}}}{4\pi }},$$

(11) $$d_{p({\mathrm{GNS}}_{1}-{\mathrm{CNT}}_{2})}=\sqrt[{3}]{\frac{3(wL_{\mathrm{GNS}}t+2Fl_{\mathrm{CNT}})}{4\pi }}.$$

where $d_{G}$ is the effective length of GNSs, *t* is the thickness of GNSs, *l* is the length of GNSs, $d_{c}$ is the effective length of CNTs, *F* is the cross-sectional area of CNTs, and $l_{re}$ is the length of fiber reinforcements.

Through calculations, the effective particle diameters of GNSs, CNTs, and GNS_1_-CNT_2_ reinforcements were found to be 121 nm, 153 nm, and 195 nm, respectively. Among them, $d_{p({GNS}_{1}-{CNT}_{2})}$ was the largest, and it was much greater than 100 nm. A larger *d_p_* means that dislocations find it more difficult to bypass the GNS_1_-CNT_2_ layer, resulting in greater dislocation pile-up and stress concentration at the interface. This provides an important prerequisite for the three-dimensional GNS_1_-CNT_2_ to bear more load transferred from the interface. Additionally, the GNS interlayer effectively prevented the stacking and agglomeration of CNTs within the CNT layers, promoting better interfacial bonding, which enhanced the role of the interface as an “intermediary” for load transfer. In summary, the three-dimensional GNS_1_-CNT_2_ reinforcement was more effective in facilitating load transfer at the interface compared to the single-structured GNSs or CNTs.

#### 4.1.2. Thermal Mismatch Strengthening

Thermal mismatch strengthening (${∆\sigma }_{CTE}$) is another important strengthening mechanism in the GNS_1_-CNT_2_/Mg composite. The contribution of dislocation strengthening induced by thermal mismatch to the yield strength of the composite can generally be expressed using the following formula [39,40]:

(12) $$∆\sigma_{\mathrm{CTE}}\;=\;\mathrm{AGb}{\left[\frac{12∆\alpha ∆TV_{f}}{bd_{G}}\right]}^{1/2}.$$

where *A* is the constant (1.25), *G* is the shear modulus of Mg (1.67 × 10^4^ MPa), *b* is the Burgers vector (3.2 × 10^−10^ m [36]), Δ*T* is the temperature difference between the sample preparation and the tensile tests, and Δ*α* is the CTE difference between pure Mg and graphene (24 × 10^−6^ K^−1^ [41]). The effective diameters of the reinforcements obtained above were substituted into Equation (12), and the calculated results are shown in Figure 9b.

As seen in Figure 9b, the primary strengthening mechanism in the GNS_1_-CNT_2_/Mg layered composite was load transfer strengthening. The theoretical calculated yield strength increments for GNS_1_-CNT_2_ reinforcements with total nanocarbon contents of 0.3 wt.% and 0.5 wt.% were 53 MPa and 77 MPa, respectively, both of which are very close to the corresponding experimental values of 51 MPa and 76 MPa. However, as the content of GNS_1_-CNT_2_ reinforcements increased, the difference between the predicted yield strength increment and the experimental result became more pronounced. When the GNS_1_-CNT_2_ content was 1.0 wt.%, the calculated yield strength increment reached 143 MPa, while the measured value was only 59 MPa. This discrepancy is due to the differences between the assumptions used in the theoretical model and the actual conditions of the composite. For example, the theoretical model assumed good interfacial bonding, whereas the increased amount of GNS_1_-CNT_2_ reinforcements easily led to stacking and agglomeration, which hindered interfacial bonding and significantly affected load transfer efficiency at the interface, resulting in a substantial deviation between the theoretical and experimental values.

### 4.2. Toughening Mechanism of Layered GNS-CNT/Mg Composites

#### 4.2.1. Effect of GNS-CNT on Dislocation Behavior

**Figure 10:**
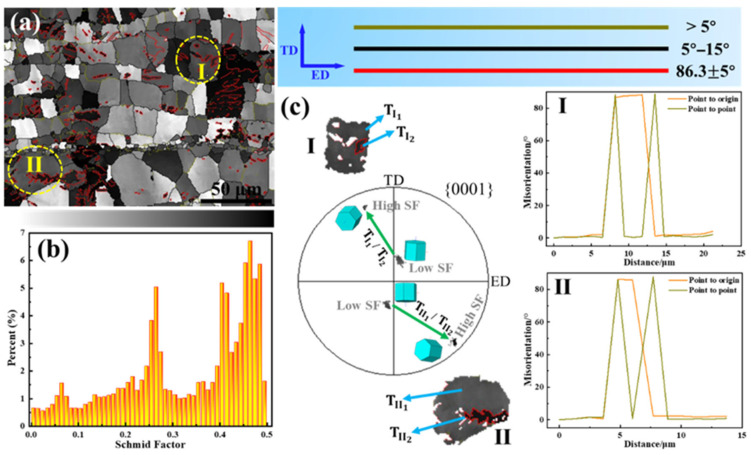
Correspondence between twinning and its texture and Schmid factors in extruded GNS_1_-CNT_2_/Mg layered composite: (**a**) Schmid distribution map, (**b**) Schmid factor distribution histogram, (**c**) Schmid factor distribution map, with the pole figure and grain boundary misorientation corresponding to the twin structure in the grain. SF: Schmid factor; TD: Transverse direction; ED: Extrusion direction; I and II: Circled grains in (**a**).

As shown in Figure 10, the introduction of nanocarbon reinforcement layers induced significant tensile twinning in the Mg matrix during hot extrusion deformation. The crystal orientation of the parent phase within the tensile twin tended towards a basal texture with a lower Schmid factor, while the twin portion exhibited an orientation away from the basal texture with a higher Schmid factor. In this way, the nanocarbon layers helped rotate the orientation of the Mg matrix grains in a direction favorable for basal slip, thereby improving the plastic deformation ability of the nanocarbon/Mg layered composite to some extent. Compared to using CNTs and GNSs as reinforcements, the unique three-dimensional network structure of the GNS_1_-CNT_2_ reinforcement resulted in a larger effective size, which generated greater constraint and local internal stress at the interface, providing more energy for tensile twinning. As a result, the GNS_1_-CNT_2_/Mg layered composite produced more tensile twins during hot extrusion deformation.

**Figure 11:**
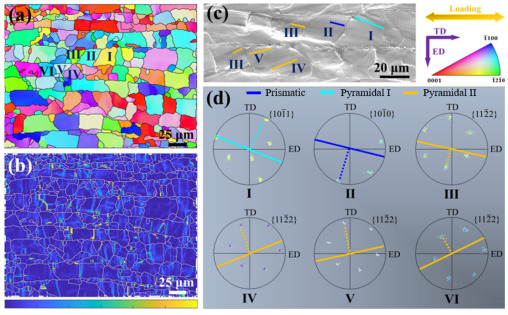
The IPF, GND density distribution, and SEM-EBSD-based slip trace analysis in the extruded GNS_1_-CNT_2_/Mg layered composite after tensile deformation: (**a**) inverse pole figure, (**b**) GND density distribution map, (**c**) high-magnification SEM image of the sample showing the slip traces, (**d**) trace analysis in different grains.

Figure 11 shows the inverse pole figure (IPF), GND density distribution, and slip trace analysis of the GNS_1_-CNT_2_/Mg layered composite after tensile deformation. Figure 11a indicates that the introduction of nanocarbon reinforcement layers did not induce abnormal growth of grains with specific orientations. The orientations of the Mg matrix grains in the composite were relatively random, without forming a specific texture. Figure 11b shows that the nanocarbon materials introduced a high geometry necessary dislocation (GND) density at the interlayer interface, which was mainly due to the deformation incompatibility between the reinforcement layer and the Mg matrix layer. The more reinforcements were added, the more GNDs needed to form in the Mg matrix to accommodate the overall strain of the material. Slip traces in six grains with high EBSD resolution were characterized (shown in Figure 11c), and the results showed that non-basal slip was activated in all six grains, with <c + a> dislocation slip successfully activated in four of them (Figure 11d). This allowed for better strain accommodation along the c-axis direction, thereby further enhancing the plastic deformation capability of the Mg matrix. Thus, the non-texturization of the GNS-CNT/Mg composites induced by the introduction of the hybrid composites led to activation of more non-basal slip and contributed to the increased ductility of the layered composites.

#### 4.2.2. Direct Toughening Effect of GNS-CNT

**Figure 12:**
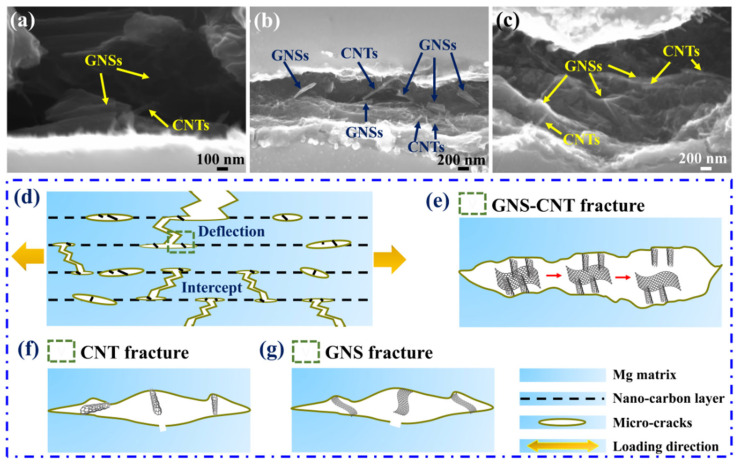
SEM fracture surfaces of the GNS-CNT/Mg layered composite during tension and a schematic diagram of failure modes for GNS-CNT, CNTs, and GNSs: (**a**–**c**) tensile fracture surfaces of GNS-CNT/Mg layered composites, (**d**) schematic illustration of the fracture mechanism in nanocarbon/Mg layered composites, (**e**–**g**) failure modes of GNS-CNT, CNTs, and GNSs at the fracture, respectively.

Figure 12 shows the in situ tensile fracture SEM images of GNS-CNT/Mg layered composites and a schematic illustration of the failure modes of the three different structures at the fracture. As can be seen, the GNS-CNT maintained its intact three-dimensional network structure at the in situ tensile fracture and remained tightly bonded to the Mg matrix side, further indicating the strong bonding between GNSs and CNTs within the GNS-CNT reinforcement, as shown in Figure 12a–c. Figure 12d–g show that all three nanocarbon layers induced a crack blunting effect, causing crack deflection and increasing the energy required for crack propagation. When CNTs or GNSs are used as reinforcements, they typically exhibit bridging across the two sides of the crack or partial pull-out at the fracture, thereby consuming energy required for crack propagation by overcoming friction with the Mg matrix during pull-out. In the three-dimensional GNS-CNT reinforcement, the strong bonding between GNSs and CNTs means that, in addition to overcoming friction with the Mg matrix during pull-out, additional energy was also needed to overcome the bonding force between GNSs and CNTs. As a result, more energy was consumed. This additional energy dissipation provided an extra toughening effect for GNS-CNT reinforcement in bridging and pull-out compared to GNSs and CNTs alone, thereby enhancing the plastic deformation resistance of the Mg matrix.

### 4.3. Electromagnetic Interference Shielding Mechanism of GNS-CNT/Mg Composites

**Figure 13:**
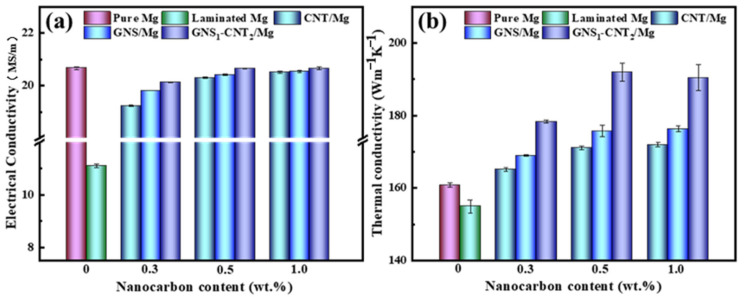
Electrical conductivity and thermal conductivity of Mg and nanocarbon/Mg layered composites: (**a**) electrical conductivity, (**b**) thermal conductivity.

Excellent electrical conductivity significantly impacts the electromagnetic shielding performance of materials [42,43]. Li et al. [44] also found that the mismatch of conductivity at the interface of PDMS, copper, and carbon nanotubes can lead to local charge accumulation and rearrangement. These non-uniform interfaces act as a place of capacitance effect, where the charge will be temporarily stored and released, thereby increasing the polarization loss and affecting the attenuation of electromagnetic waves. This not only effectively enhances the reflection loss (S_ER_) of incident electromagnetic waves at the material’s surface but also increases the multiple internal reflections of electromagnetic waves, thereby improving absorption loss (S_EA_). The influence of different nanocarbon materials on the electrical conductivity of the Mg matrix is shown in Figure 13a. It can be seen that the electrical conductivity of pure Mg was 20.67 MS/m. After introducing the layered structure, the conductivity of pure Mg decreased to 11.11 MS/m due to the presence of interlayer interfaces, representing a 46% reduction, almost halving. As the interlayer nanocarbon content increased, electrical conductivity gradually rose. When comparing different nanocarbon/Mg layered composites at the same nanocarbon content, it is evident that GNS_1_-CNT_2_/Mg exhibited higher conductivity than GNS/Mg and CNT/Mg, indicating that the interconnected three-dimensional network structure of GNS_1_-CNT_2_ provided more effective conductive pathways for free electrons, thus enhancing the overall conductivity. At a total nanocarbon content of 0.5 wt.%, the conductivities of CNT/Mg, GNS/Mg, and GNS_1_-CNT_2_/Mg were 20.31 MS/m, 20.42 MS/m, and 20.65 MS/m, respectively. When the nanocarbon content increased to 1.0 wt.%, the conductivities of these three material systems became 20.51 MS/m, 20.53 MS/m, and 20.66 MS/m, respectively, with very little increase, indicating that the conductivity of the layered composites reached saturation at 0.5 wt.% nanocarbon content.

Figure 13b shows that the thermal conductivity of the five material systems followed a trend similar to that of electrical conductivity with changing nanocarbon content. At a nanocarbon content of 0.5 wt.%, the thermal conductivities of CNT/Mg, GNS/Mg, and GNS_1_-CNT_2_/Mg were 171.08 Wm^−1^K^−1^, 175.74 Wm^−1^K^−1^, and 191.97 Wm^−1^K^−1^, respectively. When the nanocarbon content increased to 1.0 wt.%, the thermal conductivities of these three material systems became 171.98 Wm^−1^K^−1^, 176.36 Wm^−1^K^−1^, and 190.47 Wm^−1^K^−1^, respectively, with very small changes, indicating that the thermal conductivity of the Mg matrix also reached saturation at 0.5 wt.% nanocarbon content. Moreover, at the same nanocarbon content, the GNS_1_-CNT_2_/Mg composite exhibited higher thermal conductivity than GNS/Mg and CNT/Mg. This can be attributed to two reasons: first, the three-dimensional network structure of GNS_1_-CNT_2_ provided more effective thermal conduction paths for heat carriers; second, the increased effective diameter of the GNS_1_-CNT_2_ reinforcement ${\;d}_{p({\mathrm{GNS}}_{1}-{\mathrm{CNT}}_{2})}$ reduced phonon scattering at the interface, further enhancing the thermal conductivity of the composite. Yang et al.’s [45] research proved that the introduction of polydopamine can improve the interface interaction between carbon nanotubes and MXene by constructing heterostructures, thereby synergistically enhancing the electromagnetic shielding performance of materials. In addition, Xu et al. [34] also found that the multiple electromagnetic reflections of the graphene oxide multilayer structure and the interfacial polarization caused by the magnesium interlayer can significantly improve the EMI shielding performance. This is similar to our work. Higher thermal conductivity helps convert absorbed electromagnetic energy into thermal energy more quickly.

**Figure 14:**
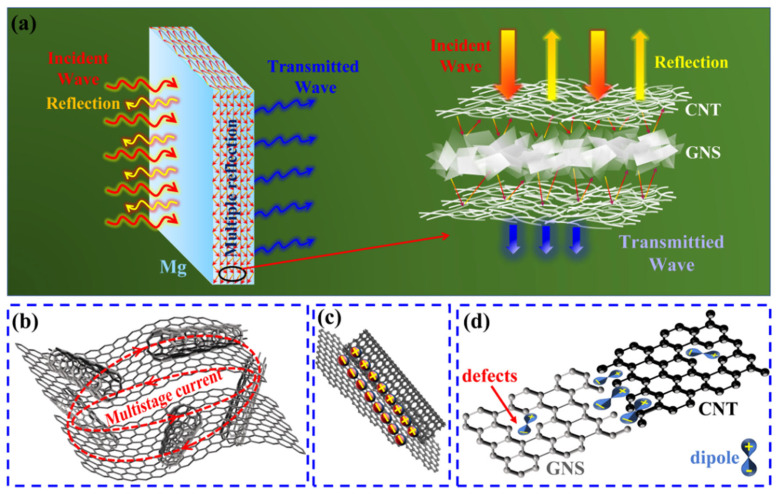
Schematic illustration of the EMI shielding mechanism of the GNS-CNT/Mg layered composites: (**a**) multiple reflection losses of incident electromagnetic waves between micro-nano layered structures, (**b**) multi−level conduction loss, (**c**) heterointerface polarization relaxation loss, (**d**) dipole polarization loss.

Figure 14 illustrates the shielding mechanism of the GNS-CNT/Mg layered composite for incident electromagnetic waves. First, when the incident electromagnetic waves contacted the surface of the composite, part of the waves were directly reflected, resulting in reflection loss (S_ER_). The remaining waves that entered the interior of the composite underwent multiple reflections between the micro-nano layers, extending the transmission path of the waves within the composite and effectively increasing the absorption loss (S_EA_) of the material [46]. The three-dimensional network structure of the GNS-CNT reinforcement further helped mitigate the impedance mismatch between the Mg micro-layers and the nanocarbon layers, allowing the incident electromagnetic waves to penetrate more deeply into the nanocarbon material. Additionally, the complex three-dimensional network in the GNS-CNT reinforcement created numerous contact interfaces between the GNSs and CNTs, and these multiple interfaces facilitated multistage reflection and scattering of the incident electromagnetic waves. This extended the transmission path of the waves within the GNS-CNT layer, thus further improving the absorption loss (S_EA_) of the GNS-CNT layer, as shown in Figure 14a. Secondly, the material generated a time-varying long-range induced current under an alternating electromagnetic field. As previously analyzed, compared to GNSs and CNTs with the same content, GNS-CNT exhibited higher electrical conductivity due to its unique three-dimensional network structure. This comprehensive three-dimensional multistage conductive network in GNS-CNT extended the migration path of free electrons, allowing the long-range induced current to be rapidly converted into heat through ohmic loss within the three-dimensional resistive network, causing the incident electromagnetic wave energy to be quickly attenuated in the nanocarbon layers. This promoted the absorption and energy conversion of electromagnetic waves by the GNS-CNT layer, as shown in Figure 14b.

Furthermore, compared to GNS/Mg and CNT/Mg, the GNS-CNT/Mg layered composite not only had interfaces between the reinforcements and the Mg matrix but also possessed heterogeneous interfaces between GNSs and CNTs within the complex three-dimensional GNS-CNT network. When the incident electromagnetic wave entered the GNS-CNT reinforcement, under the influence of the alternating electromagnetic field, free charges tended to accumulate and oscillate at the GNS and CNT heterogeneous interfaces, leading to interfacial polarization and the induction of macroscopic dipole moments that further promoted the attenuation of incident electromagnetic waves through polarization relaxation [47], as shown in Figure 14c. Finally, under an alternating electromagnetic field, high-density defects such as the numerous exposed edge sites of GNSs and CNTs, dislocations at heterogeneous interfaces, and lattice distortions at connection nodes within the three-dimensional GNS-CNT network generated localized electric dipoles that reacted with the incident electromagnetic waves. Dipole polarization further enhanced the absorption loss of electromagnetic waves of the GNS-CNT layer. Notably, after acid treatment of GNSs and CNTs, a large number of oxygen-containing functional groups remained on the GNS-CNT surface. Due to the significant difference in attracting electrons between oxygen and carbon atoms, these functional groups also served as polarization centers, contributing to the attenuation of incident electromagnetic waves to some extent, as shown in Figure 14d. Stupar et al. [48] found that the higher the mass fraction of metal silver ions deposited on graphite, the better the electromagnetic shielding performance. They determined that this may be because the number of random collisions between the reflected wave and these particles increases, resulting in a further weakening of the power of the electromagnetic beam, thereby improving the shielding performance of the material. Based on the above analysis, the GNS-CNT/Mg layered composite exhibited higher electromagnetic shielding effectiveness compared to GNS/Mg and CNT/Mg.

## 5. Conclusions

This study investigated the effect of graphene nanosheet (GNS) and carbon nanotube (CNT) hybrid reinforcements on the mechanical properties and electromagnetic shielding performance of Mg-based composites. The GNS-CNT hybrid reinforcement was specifically analyzed for its structural advantages and effectiveness compared to single-component reinforcements. Based on the experimental results, the following conclusions are drawn:

1. The GNS-CNT hybrid reinforcement significantly improved both the mechanical properties and electromagnetic shielding effectiveness of the Mg-based composite. At a nanocarbon content of 0.5 wt.% with a GNS-CNT ratio of 1:2, the composite achieved optimal performance, with tensile strength increased by up to 55% and total electromagnetic shielding effectiveness (S_ET_) reaching 70 dB, outperforming composites reinforced with individual CNTs or GNSs.
2. The GNS_1_-CNT_2_ hybrid reinforcement provided a more effective load transfer mechanism compared to individual GNSs or CNTs. The three-dimensional network structure generated greater constraint and local internal stress at the interface, inducing more tensile twinning during deformation and effectively improving the plastic deformation capability and overall mechanical strength of the composite.
3. The unique three-dimensional network of GNS_1_-CNT_2_ facilitated multiple internal reflections and better impedance matching, enhancing the absorption loss of incident electromagnetic waves. This, along with high electrical conductivity, resulted in a more efficient conversion of electromagnetic energy into heat, providing superior electromagnetic shielding compared to CNTs or GNSs alone.

## Figures and Tables

**Table 1:** Chemical composition of initial Mg foil [6].

Element	Fe	Si	Ni	Cu	Al	Cl	Mn	Ti	Mg
Content (wt.%)	0.004	0.005	0.0007	0.003	0.006	0.003	0.010	0.014	0.9543

**Table 2:** Mechanical properties of pure Mg and nanocarbon/Mg layered composites.

Samples	YS (MPa)	UTS (MPa)	Elongation (%)
Pure Mg	89 ± 3	158 ± 6	6.4 ± 0.2
Layered Mg	93 ± 2	187 ± 4	8.3 ± 0.3
0.3 wt.% CNT/Mg	123 ± 4	204 ± 3	9.2 ± 0.6
0.3 wt.% GNS/Mg	122 ± 6	202 ± 4	12.0 ± 0.4
0.3 wt.% GNS_1_-CNT_2_/Mg	140 ± 5	224 ± 8	13.1 ± 0.7
0.5 wt.% CNT/Mg	128 ± 2	214 ± 3	10.1 ± 0.9
0.5 wt.% GNS/Mg	125 ± 3	210 ± 5	10.8 ± 0.5
0.5 wt.% GNS_1_-CNT_2_/Mg	142 ± 4	224 ± 7	11.0 ± 0.4
0.5 wt.% GNS_1_-CNT_2_/Mg	165 ± 3	245 ± 4	11.5 ± 0.8
0.5 wt.% GNS_1_-CNT_4_/Mg	133 ± 5	222 ± 3	10.9 ± 0.5
1.0 wt.% CNT/Mg	130 ± 4	195 ± 5	4.5 ± 1.1
1.0 wt.% GNS/Mg	123 ± 7	187 ± 4	4.2 ± 0.6
1.0 wt.% GNS_1_-CNT_2_/Mg	148 ± 6	223 ± 5	8.1 ± 0.7
0.3 wt.% GNS_1_-CNT_2_/Mg	140 ± 5	224 ± 8	13.1 ± 0.7

**Table 3:** Comparison of SE (X-band: 8.2–12.4 GHz) in this work with previously reported materials.

Materials	Mass Fraction (wt. %)	SE(dB)	Reference
GNS_1_-CNT_2_/Mg composite	0.5	70	this work
CNTs/Mg	0.5	58	[6]
GNSs/Mg	0.5	55	[7]
Mg/15Fe	15	55	[28]
6NZCF/Mg-9Li	6	58	[29]
Ni/Ag	14	47	[30]
MWCNT/PANI	25	39	[31]
SWCNT/PU	20	17	[32]
(SLG-FLG)/EP	4	30	[12]

**Table 4:** Comparison of SE (X-band: 8.2–12.4 GHz) and mechanical properties in this work with previously reported materials.

Materials	SE(dB)	YS (MPa)	UTS (MPa)	Elongation (%)	Reference
0.50 wt% GNS_1_-CNT_2_/Mg composite	70	168	249	12.3	this work
0.50 wt% CNTs/Mg	58	130	213	12.1	[6]
0.50 wt% GNSs/Mg	55	125	216	12.3	[7]
PDA@HGM_5_	49.8	-	32.31	-	[33]
GO/AZ31	23.8	230	290	13.6	[34]
GO-SnO_2_/AZ31	30	266	310	13.2	[34]

## Data Availability

The original contributions presented in this study are included in the article. Further inquiries can be directed to the corresponding authors.
